# The Safety and Efficacy of the Early Use of Sacubitril/Valsartan After Acute Myocardial Infarction: A Meta-Analysis of Randomized Controlled Trials

**DOI:** 10.7759/cureus.53784

**Published:** 2024-02-07

**Authors:** Abdullah -, Majid Rashid, Cuauhtemoc Jeffrey Soto, Ghazala S Virk, Favour C Mekowulu, Sandipkumar S Chaudhari, Saima Batool, Muhammad Usama

**Affiliations:** 1 Kidney Transplant Unit, Rehman Medical Institute, Peshawar, PAK; 2 Internal Medicine, Khyber Teaching Hospital (KTH) Medical Teaching Institute, Peshawar, PAK; 3 Research and Development, Universidad Juarez del Estado de Durango, Durango, MEX; 4 Internal Medicine, Avalon University School of Medicine, Youngstown, USA; 5 Internal Medicine, V.N. Karazin Kharkiv National University, Kharkiv, UKR; 6 Cardiothoracic Surgery, University of Alabama at Birmingham, Birmingham, USA; 7 Family Medicine, University of North Dakota School of Medicine and Health Sciences, Fargo, USA; 8 Internal Medicine, Hameed Latif Hospital, Lahore, PAK; 9 Neurology, Sheikh Zayed Hospital, Rahim Yar Khan, PAK

**Keywords:** randomized controlled trials, meta-analysis, acute myocardial infarction, sacubitril/valsartan, early use, efficacy, safety

## Abstract

Acute myocardial infarction (AMI) is a significant global cause of mortality, necessitating the exploration of innovative treatments against the condition. Angiotensin receptor blockers (ARBs), angiotensin-converting enzyme inhibitors (ACEIs), and angiotensin receptor-neprilysin inhibitors (ARNIs) such as sacubitril/valsartan have demonstrated promise in managing acute heart failure (HF). However, despite favorable evidence from clinical trials for the use of sacubitril/valsartan in AMI, its overall efficacy remains a subject of debate. Hence, we conducted this review and meta-analysis, by adhering to the Preferred Reporting Items for Systematic Reviews and Meta-Analysis (PRISMA) guidelines and aligned with European Society of Cardiology recommendations, to compare sacubitril/valsartan with traditional ACEI/ARB treatments for AMI. We employed Review Manager 5.4 for statistical analysis, the Risk of Bias Tool 2.0 was utilized for quality assessment, and publication bias was assessed using a funnel plot. A p-value <0.05 was considered statistically significant.

Eight randomized controlled trials (RCTs) were included in this meta-analysis. Our findings revealed that participants treated with sacubitril experienced significantly improved outcomes in terms of HF (OR=0.79; 95% CI: 0.66-0.95; p=0.01; I^2^=23%), N-terminal pro-B-type natriuretic peptide (NT-proBNP) levels (MD = -1.58; 95% CI: -1.78 to -1.37, p<0.00001; I^2^=97%), and major adverse cardiovascular events (MACE) (OR=0.84; 95% CI: 0.72-0.99; p=0.03; I^2^=44%). However, left ventricular ejection fraction (LVEF) (MD=3.68; 95% CI: 3.35-4.01, p<0.00001; I^2^=71%) showed greater improvement in the control group compared to the experimental group.

Our meta-analysis suggests that sacubitril offers a favorable balance between safety and effectiveness. Sacubitril significantly improved outcomes in terms of HF, MACE, and NT-proBNP levels when compared to the control group. However, improvement in LVEF was notably higher in the control group over the sacubitril/valsartan group.

## Introduction and background

Acute myocardial infarction (AMI), commonly known as a heart attack, arises from the total occlusion of a coronary artery, halting blood supply to cardiomyocytes or due to demand ischemia in a non-obstructed coronary artery [1]. Myocardial ischemia, an oxygen supply-demand imbalance, precedes MI and is diagnosed based on patient history, ECG, and elevated biomarkers. The symptoms include chest, upper limb, mandibular, or epigastric discomfort, with dyspnea and exhaustion [2]. Retrosternal chest pain, feeling like pressure, may occur intermittently or persistently, radiating to the left shoulder, neck, or upper extremities [3]. AMI is associated with the loss of around one million lives in the United States annually, with a global impact affecting approximately three million individuals [4]. Despite notable advancements in therapy, AMI continues to be a prominent cause of mortality [5].

The main objective of percutaneous coronary intervention (PCI) is to improve blood flow to the ischemic area and relieve constriction or blockage of the coronary artery [6]. The efficacy of this intervention in managing AMI has been recognized, based on its ability to target coronary artery stenosis, chest discomfort, and related clinical symptoms [7-9]. Nevertheless, it is worth noting that there is a high incidence of postoperative problems, such as stent thrombosis and the need for target lesion blood transport reconstruction, which have been associated with increased death rates [10,11]. Also, left ventricular dysfunction is a prominent contributor to mortality among hospitalized patients, regardless of the patient population, especially in cases where initial PCI revascularization is unsuccessful [12].

Advancements in medical treatment have led to a significant reduction in overall and cardiovascular mortality rates, along with reduced hospitalizations for heart failure (HF) and other causes [13]. However, heart dysfunction after surgery persists despite technical advances. Studies in the literature have highlighted the advantages associated with the utilization of angiotensin-converting enzyme inhibitors and angiotensin receptor blockers (ACEIs/ARBs) to enhance patient outcomes [14-17]. ACEIs have demonstrated efficacy in improving the survival rates of patients with congestive HF and asymptomatic left ventricular systolic dysfunction following MI. In addition, the aforementioned interventions have served to reduce the intensity and frequency of MIs as evidenced by several scholarly sources [18-20]. According to a seminal study conducted in 1986, the timely administration of intravenous lytic medicines to individuals experiencing MI resulted in a significant (50%) decrease in mortality rates [21]. This gave rise to the notion of "the golden hour," which posits that prompt thrombolysis could potentially avert mortality. Rapid revascularization techniques have been shown to provide substantial benefits for individuals diagnosed with ST-segment elevation myocardial infarction (STEMI) [22].

A better understanding of the mechanisms that underlie HF and cardiac remodeling has facilitated the development of innovative therapeutic strategies. The advent of angiotensin receptor-neprilysin inhibitors (ARNIs) represents a noteworthy advancement in the field [23,24]. Regulatory approval has been granted to sacubitril/valsartan, which is the pioneering drug within its respective category. Sacubitril is classified as a pro-drug that, upon undergoing activation, functions as a neprilysin inhibitor. The mechanism of action involves the inhibition of neprilysin, impeding the degradation of natriuretic peptides. Consequently, this results in an extended duration of the beneficial effects exerted by these peptides [25]. In the context of HF therapy, it is advisable to consider the utilization of sacubitril/valsartan as an alternative to ACEIs or ARBs. This recommendation is made in conjunction with the concurrent administration of other frequently prescribed medications such as beta-blockers and aldosterone antagonists [9,26]. Research has provided evidence that sacubitril-valsartan outperforms ARBs in terms of lowering HF exacerbations that result in hospitalizations or urgent ambulatory visits. Additionally, there is a positive correlation between a lower left ventricular ejection fraction (LVEF) and greater rates of survival [23].

Although clinical trials have demonstrated potential in the early administration of sacubitril/valsartan for AMI, there exists a variety of viewpoints regarding its overall effectiveness. In light of this, to create a strong scientific basis for its use, a comprehensive analysis of randomized controlled trials (RCTs) was conducted to specifically investigate the effects of sacubitril/valsartan in patients with AMI. The primary objective of this analysis was to provide practical guidance for the utilization of sacubitril/valsartan in the treatment of AMI. This meta-analysis thoroughly examined RCTs on early sacubitril/valsartan administration in AMI. Our findings strongly support its efficacy, revealing benefits such as lowered heart rate, improved ejection fraction, reduced N-terminal pro-B-type natriuretic peptide (NT-proBNP), and minimized major adverse cardiovascular events (MACE) in AMI patients with HF. Our findings underscore that initiating sacubitril/valsartan promptly after AMI is both safe and effective.

## Review

Methods

This meta-analysis was performed in compliance with the Preferred Reporting Items for Systematic Reviews and Meta-Analysis (PRISMA) guidelines [27].

Search Strategy

Two authors conducted searches involving several databases, including Cochrane, PubMed, Embase, and Medline for articles, spanning the time from their initiation till October 5, 2023. The database searches were conducted by using a combination of the following terms: "Myocardial Infarction, Neprilysin Inhibitor, LCZ696, Sacubitril/Valsartan, and Entresto." There were no limitations placed on the use of language or any temporal constraints. The search approach is summarized in Table 1.

**Table 1:** Search strategy* *[1-52]

Database	Query	Search details	Number of patients
PubMed	Myocardial Infarction AND Neprilysin Inhibitor OR LCZ696 OR Sacubitril/Valsartan OR Entresto	(("myocardial infarction"[MeSH Terms] OR ("myocardial"[All Fields] AND "infarction"[All Fields]) OR "myocardial infarction"[All Fields]) AND (("neprilysin"[MeSH Terms] OR "neprilysin"[All Fields] OR "neprilysins"[All Fields]) AND ("antagonists and inhibitors"[MeSH Subheading] OR ("antagonists"[All Fields] AND "inhibitors"[All Fields]) OR "antagonists and inhibitors"[All Fields] OR "inhibitors"[All Fields] OR "inhibitor"[All Fields] OR "inhibitor s"[All Fields]))) OR ("sacubitril and valsartan sodium hydrate drug combination"[Supplementary Concept] OR "sacubitril and valsartan sodium hydrate drug combination"[All Fields] OR "lcz696"[All Fields]) OR ("sacubitril and valsartan sodium hydrate drug combination"[Supplementary Concept] OR "sacubitril and valsartan sodium hydrate drug combination"[All Fields] OR "sacubitril valsartan"[All Fields]) OR ("sacubitril and valsartan sodium hydrate drug combination"[Supplementary Concept] OR "sacubitril and valsartan sodium hydrate drug combination"[All Fields] OR "entresto"[All Fields] OR "sacubitril"[Supplementary Concept] OR "sacubitril"[All Fields] OR "valsartan"[MeSH Terms] OR "valsartan"[All Fields])	5,882
Embase	Neprilysin Inhibitor OR LCZ696 OR Sacubitril/Valsartan OR Entresto		903
Cochrane Library	Neprilysin Inhibitor OR LCZ696 OR Sacubitril/Valsartan OR Entresto		434
SCOPUS	Neprilysin Inhibitor OR LCZ696 OR Sacubitril/Valsartan OR Entresto		783
Google Scholar	Neprilysin Inhibitor OR LCZ696 OR Sacubitril/Valsartan OR Entresto		1,423

Inclusion and Exclusion Criteria

The inclusion criteria were as follows: (I) studies including patients with MI, (II) studies including patients administered sacubitril/valsartan in the experimental group, (III) RCTs, and (IV) studies that provide outcomes of interest. The exclusion criteria were as follows: (I) non-RCTs, (II) studies that do not provide efficacy-related data, and (III) in cases where multiple publications on the same clinical research were found, only the publication with the most thorough data were considered eligible.

Data Extraction and Outcome Measures

The data were extracted by two researchers. The extracted information included the study title, author, publication year, baseline characteristics of the subjects (gender and age), source of research subjects, and sample size. The primary outcomes of our study were MACE and HF, while secondary outcomes included LVEF and NT-proBNP.

Quality Assessment and Statistical Analysis

**Figure 1:**
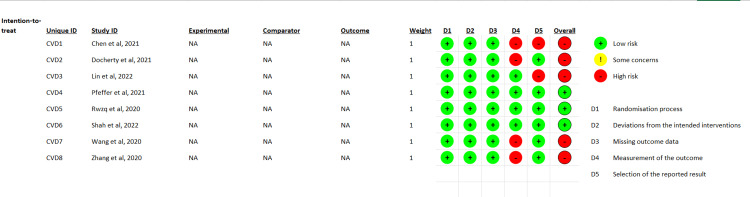
Quality assessment

**Figure 2:**
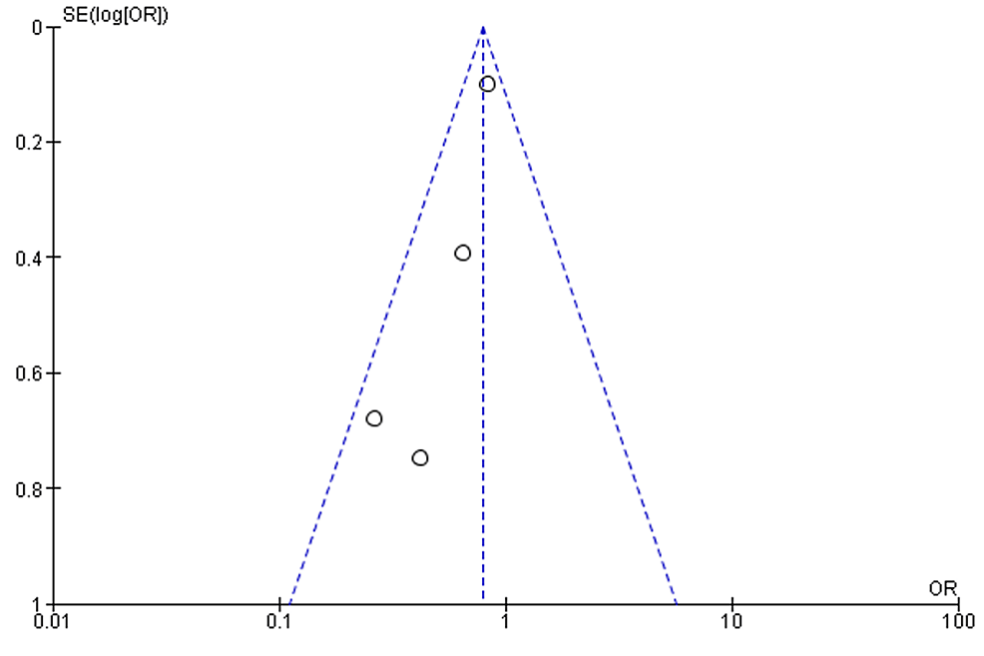
Funnel plot for the outcome of HF HF: heart failure

One researcher was tasked with using the Risk of Bias Tool 2 (ROB 2.0) to assess quality (Figure 1). Forest plots and statistical analysis were completed using Review Manager 5.4. The pooled effect size was calculated using forest plots by employing either random or fixed effects. The researchers employed a fixed-effects model in cases when the value of I^2^ was below 50%. Conversely, a random-effects model was utilized when I^2^ exceeded 50%. The evaluation of publication bias was conducted by employing a funnel plot, as depicted in Figure 2. The determination of the significance threshold (p<0.05) was made based on the Z value.

Results

Literature Search Results

**Figure 3:**
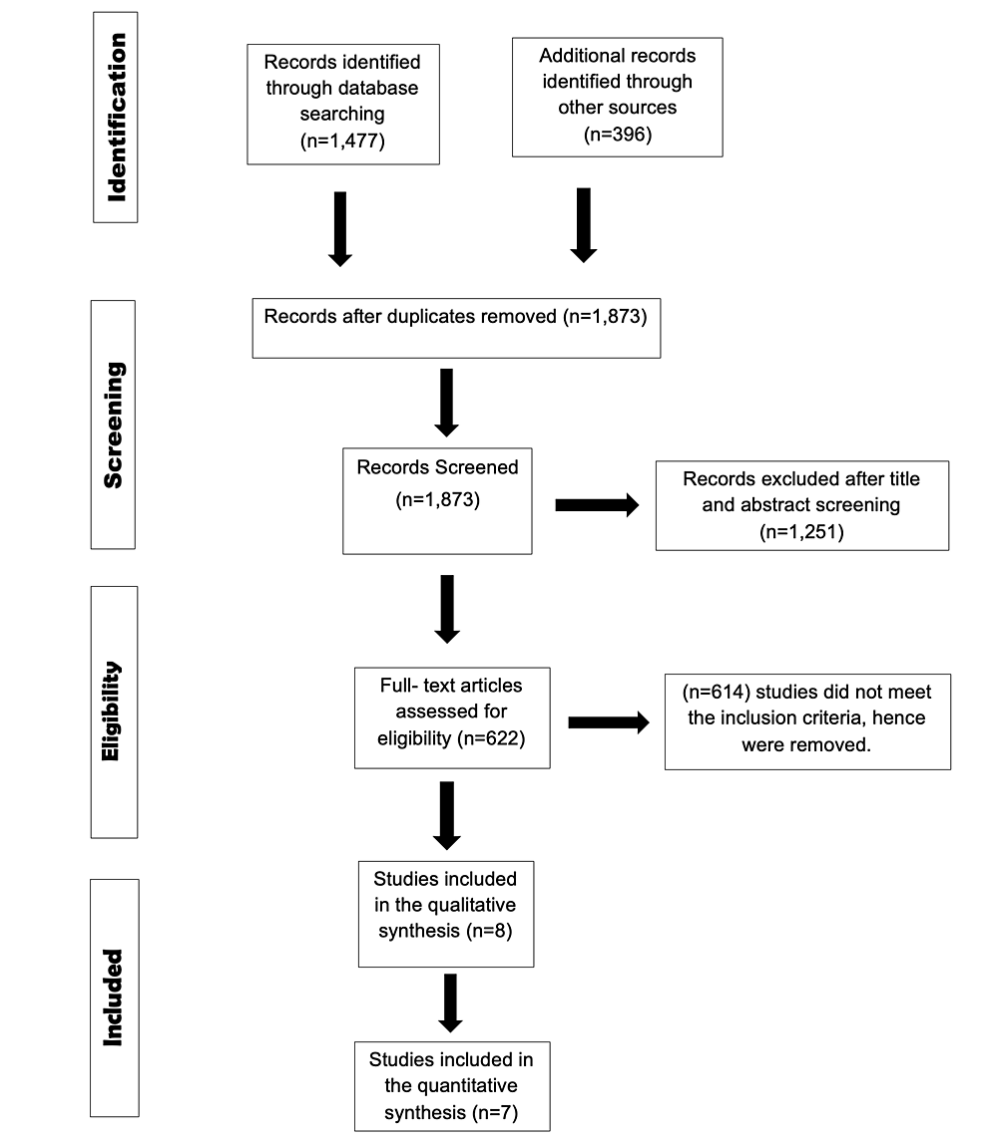
PRISMA flow diagram illustrating the selection of studies PRISMA: Preferred Reporting Items for Systematic Reviews and Meta-Analysis

Figure 3 presents the PRISMA flow chart, which visually outlines the extensive screening procedure. After eliminating duplicate entries and implementing filters, a total of eight papers were selected for the final analysis.

Baseline Characteristics

Table 2 provides an overview of the characteristics of the included studies and the demographic information of the participants. The studies involved 6,981 participants, with a mean age of 59.4 years in the experimental group and 59.2 years in the control group. The experimental group consisted of 76.65% males.

**Table 2:** Baseline characteristics of the included studies* *[45-52] ACEI: angiotensin-converting enzyme inhibitor

Author	Year	Disease	Sample size		Age, years, mean ±SD		Sex, male/total		Intervention	
			Experimental	Control	Experimental	Control	Experimental	Control	Experimental	Control
Chen et al. [45]	2021	Acute myocardial infarction	42	39	51.28 ±6.27	51.3 ±6.21	27/42	24/39	Sacubitril/valsartan	Bisoprolol
Docherty et al. [46]	2021	Myocardial infarction	47	46	61.8 ±10.6	59.7 ±10.1	42/47	43/46	Sacubitril/valsartan	Valsartan
Rezq et al. [47]	2020	ST-segment elevation myocardial infarction	100	100	52 ±9.2	57 ±11.6	86/100	88/100	Sacubitril/valsartan	Ramipril
Wang et al. [44]	2020	Acute anterior wall myocardial infarction	68	69	59.13 +7.15	60.56 ±7.62	52/68	54/69	Sacubitril/valsartan	Enalapril
Zhang et al. [49]	2020	ST-elevation myocardial infarction	79	77	60.3 ±11.7	60 ±10.9	59/79	55/77	Sacubitril/valsartan	ACEI
Pfeffer et al. [50]	2021	Myocardial infarction	2,830	2,831	64.0 ±11.6	63.5 ±11.4	2167/2830	2131/2831	Sacubitril/valsartan	Ramipril
Lin et al. [51]	2022	Acute anterior wall ST-elevation myocardial infarction	55	54	61.38 ±12.31	59.74 ±11.53	49/55	47/54	Sacubitril/valsartan	Valsartan
Shah et al. [52]	2022	Acute myocardial infarction	279	265	65.0 ±11.9	62.3 ±11.2	201/279	201/265	Sacubitril/valsartan	Ramipril

Results of the Meta-Analysis

**Figure 4:**
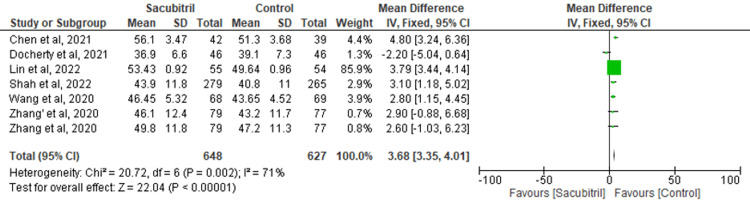
Forest plot for the outcome of LVEF LVEF: left ventricular ejection fraction

**Figure 5:**
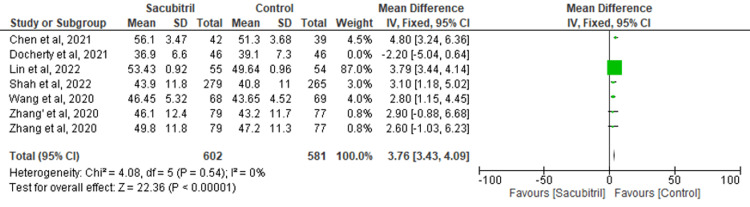
Sensitivity analysis for the outcome of LVEF LVEF: left ventricular ejection fraction

LVEF: Data from six studies were pooled to evaluate the effect of sacubitril on the outcome of LVEF. It was found that there was a significant improvement in LVEF in the control group versus the experimental group (MD=3.68; 95% CI: 3.35-4.01, p<0.00001; I^2^=71%) (Figure 4). To address the heterogeneity in the results, a sensitivity analysis was conducted. Upon excluding the study by Docherty et al., the results remained significant (MD=3.76; 95% CI: 3.43-4.09, p<0.00001), but the heterogeneity lowered to 0% (Figure 5).

**Figure 6:**
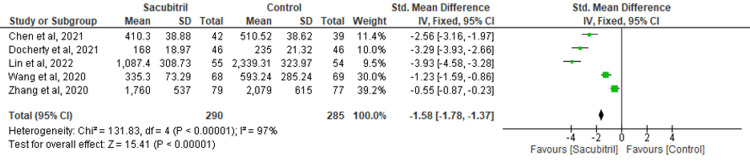
Forest plot for the outcome of NT-proBNP NT-proBNP: N-terminal pro–B-type natriuretic peptide

NT-proBNP: Five of the included studies assessed the effectiveness of sacubitril versus control for the outcome of NT-proBNP. The outcome of NT-proBNP was improved in the group of sacubitril as compared to the control group (MD = -1.58; 95% CI: -1.78 to -1.37, p<0.00001; I^2^=97%) (Figure 6).

**Figure 7:**
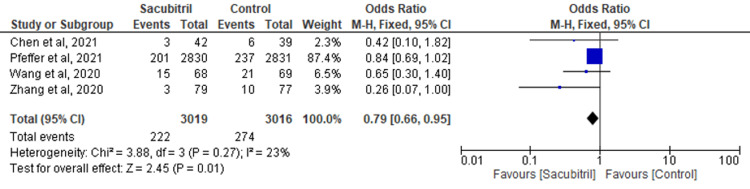
Forest plot for the outcome of HF HF: heart failure

HF: The findings of four RCTs were combined to evaluate the outcome of HF. The results revealed that the participants taking sacubitril had a lower incidence of HF when compared to the control group (OR=0.79; 95% CI: 0.66-0.95; p=0.01; I^2^=23%) (Figure 7).

**Figure 8:**
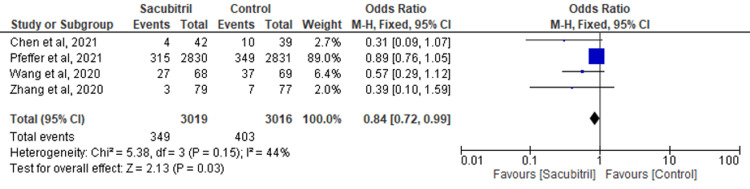
Forest plot for the outcome of MACE MACE: major adverse cardiovascular events

MACE: Four studies examined the efficacy of sacubitril in comparison to a control group concerning the occurrence of MACE as the outcome. The findings indicated that individuals who received sacubitril experienced a reduced occurrence of MACE in comparison to the control group (OR=0.84; 95% CI: 0.72-0.99; p=0.03; I^2^=44%) (Figure 8).

Discussion

In determining the impact of sacubitril on HF after MI, our meta-analysis revealed that the drug was successful in enhancing the outcomes of HF, NT-proBNP, and MACE, yielding significant results when compared to the control group. However, the improvement in the outcome of LVEF was greater in the control group versus the experimental group. A sensitivity analysis was performed to account for the heterogeneity in LVEF outcome results, and it was found that the results remained consistent, and heterogeneity was significantly reduced.

Several previous meta-analyses have been conducted on the effect of sacubitril on HF outcomes. A study by Liu et al. [28] reported similar results to our findings. This study highlighted the effectiveness of sacubitril in improving the outcome of HF (MD=0.49, 95% CI: 0.27-0.89, I^2^=0%, p=0.02), NT-proBNP (MD = -1.33%, 95% CI: -1.54 to -1.12, I^2^=96%, p<0.00001), and MACE (MD=0.49, 95% CI: 0.27-0.89, I^2^=0%, P=0.02). However, this research included only five RCTs, while our study encompassed seven RCTs, thereby reinforcing the strength of the evidence presented in this study. Xiong et al. [29] showed significant improvement in NT-proBNP (WMD = -310.23, 95% CI: -385.89 to -234.57, p<0.001), which aligns with the results of our meta-analysis. However, this study showed an improvement in LVEF (WMD=5.49, 95% CI: 3.62-7.36, p<0.001) in the sacubitril group, whereas our findings favored the control group. In line with our results, Zhao et al. [30] demonstrated a reduction in MACE among participants taking sacubitril (RR: 0.61, 95% CI: 0.46-0.82, P=0.001). In contrast with our findings, a study by Nie et al. [31] failed to show any significant difference between ACEIs and sacubitril for the outcomes of NT-proBNP (WMD = -301.16; 95% CI: -602.77-0.437; p=0.05) and LVEF (WMD: 1.49; 95% CI: -1.33-4.32; p=0.30).

The pathophysiology of HF involves a maladaptive response characterized by an activated renin-angiotensin-aldosterone system (RAAS), leading to vasoconstriction, hypertension, increased aldosterone levels, heightened sympathetic tone, and ultimately, cardiac remodeling, all of which contribute to the progression of the disease [32]. In addition to impeding neprilysin, sacubitril concurrently blocks the AT1 receptor when combined with valsartan. Together, these two effects decrease aldosterone and increase natriuretic peptides. These results support the use of sacubitril/valsartan to control fluid balance in treating cardiovascular illnesses [33]. Sacubitril's enhanced NPs improve hemodynamics when the ejection fraction is lower. They lower levels of renin, aldosterone, and vasopressin, increase natriuresis, and inhibit the sympathetic nervous system. They also lower ventricular filling pressures in severe heart failure, primarily affecting preload without altering systemic vascular resistances [34]. To lower afterload and raise LVEF, combining neprilysin inhibition with RAAS inhibition is recommended.

Heart-secreted neuroendocrine hormones - BNP and NT-proBNP - have proven to be useful markers for the diagnosis, management, and prognosis of HF patients [35,36]. The measurement of the size of MI, the therapeutic effect of myocardial reperfusion, and the anticipation of myocardial remodeling are all significantly influenced by NT-proBNP [37]. Changes in NT-proBNP levels have been shown to correlate highly with cardiovascular outcomes in patients with heart failure with reduced ejection fraction (HFrEF). When it comes to lowering NT-proBNP, sacubitril/valsartan is noticeably better than enalapril, highlighting its potential to lower morbidity and mortality. The fact that NT-proBNP is still a valid indicator of HF severity in the presence of neprilysin inhibition highlights the significance of this biomarker in determining the efficacy of treatment [38].

The main pathological cardiac remodeling is caused by the excessive stimulation of RAAS and sympathetic nerves [10,11]. Sacubitril inhibits RAAS by improving glomerular filtration rate and renal blood flow. Additionally, it provides anti-hypertrophic and anti-fibrotic benefits [39] and volume reduction [40]. MACE in patients with AMI after PCI poses a risk to the quality of life and health. A precise prediction of the risk of MACE following PCI enables the early implementation of appropriate measures for control and prevention, thereby reducing the occurrence of MACE [41].

The adverse effects include angioedema, renal failure, hyperkalemia, hypotension, and cough [42]. Sacubitril/valsartan increases hypotension and symptomatic hypotension. However, sacubitril/valsartan is associated with a reduced risk of cough and potassium/creatinine increase than enalapril. Angioedema has also been found to be more common with sacubitril/valsartan than enalapril [43]. Sacubitril/valsartan failed to improve LVEF in our study, and this could be attributed to study heterogeneity and publication bias. Understanding these limitations is crucial in evaluating the effectiveness of sacubitril/valsartan in enhancing LVEF and tailoring treatment strategies for optimal outcomes.

Sacubitril's use in the treatment of HF has significant implications for both clinical practice and research. Sacubitril has been demonstrated to enhance several important HF-related parameters, making it a compelling therapeutic alternative. Firstly, it has been effective in raising NT-proBNP levels, an important biomarker for the severity and prognosis of HF. Improved outcomes and enhanced heart function are indicated by lower NT-proBNP levels. Furthermore, sacubitril has demonstrated its capacity to raise LVEF, a critical measure of heart health. Sacubitril improves cardiac function and HF patients' overall quality of life [44] by raising LVEF. Additionally, sacubitril therapy's correlation with a decrease in MACE presents a significant benefit in terms of patient safety and long-term results. This finding highlights sacubitril's ability to lower the likelihood of adverse effects, including cardiovascular mortality, hospitalizations, and other complications.

From a scientific perspective, research on sacubitril's effect on HF outcomes advances both our knowledge of its mechanisms and the creation of more potent HF medicines. The usefulness of sacubitril in contemporary HF care is further supported by the consistent findings across several clinical investigations. However, further studies are required to fully understand the subtleties of its effects, long-term advantages, and possible combinations with other therapeutic approaches. To conclude, sacubitril's beneficial effects on NT-proBNP, LVEF, HF, and MACE signify a hopeful advancement in cardiovascular medicine that could greatly enhance the quality of life for patients who suffer from HF and improve our understanding of the treatment of HF.

This meta-analysis has a few limitations. The research included in the analysis exhibited a generally moderate degree of quality, and the sample sizes were considered inadequate. Certain papers did not explicitly mention the utilization of certain randomized procedures, while others failed to provide precise details regarding the pharmacological specifics pertaining to Western conventional drug treatment. Furthermore, the publications exhibited clinical heterogeneity due to variations in the age groups of the research participants and the coexistence of comorbidities. The funnel plot in our meta-analysis showed an element of publication bias. This potential bias could impact the robustness and generalizability of our findings. Our dedication to a responsible and thorough research approach is reinforced by our transparency in identifying limitations. Readers must take these elements into account while evaluating the data.

## Conclusions

Based on our findings, the use of sacubitril appears to offer a favorable balance between safety and effectiveness. The outcomes of HF, MACE, and NT-proBNP showed significant improvement with sacubitril use when compared to the control group. However, the improvement in LVEF was significantly greater in the control group.
